# Efficacy of three different techniques in the fluoroscopy-guided intra-articular steroid injection of the hip: a randomized controlled trial

**DOI:** 10.1038/s41598-023-44595-5

**Published:** 2023-10-11

**Authors:** Rakop Taveesuksiri, Prapasri Kulalert, Chane Jitapunkul, Adinun Apivatgaroon

**Affiliations:** 1 Department of Orthopaedics, Faculty of Medicine, Thammasat University, Pathum Thani, Thailand; 2 Department of Clinical Epidemiology, Faculty of Medicine, Thammasat University, Pathum Thani, Thailand

**Keywords:** Health care, Medical research

## Abstract

Fluoroscopy-guided injection via the anterior (A), anterolateral (AL), or proximal anterolateral (PAL) approaches are the common hip injection techniques without comparing the efficacy of the three techniques. The prospective randomized controlled trial was conducted from August 2020 to March 2022. Included patients with intra-articular hip disorders indicated an intra-articular steroid injection. Excluded significant spine pathology with radiculopathy or significant neurological deficits, previous hip surgery of the injection side, suspected tumor or infection origins, steroid or contrast media allergy, and body mass index > 35 kg/m^2^. The primary outcome was the injection attempt defining one attempt and multiple attempts. 90 patients were recruited and allocated to 30 per group. There were no differences between A, AL, and PAL respectively regarding the success in one attempt rate (80%, 80%, 90%; p = 0.533), VAS during local anesthetic injection (4.33 ± 1.99, 3.70 ± 2.34, 4.27 ± 2.49; p = 0.500), VAS during intra-articular injection (4.27 ± 1.87, 4.70 ± 2.37, 4.13 ± 2.37; p = 0.587), radiation doses (0.558 ± 0.313, 0.526 ± 0.485, 0.492 ± 0.275 mGy; p = 0.788), radiation time (0.043 ± 0.017, 0.039 ± 0.021, 0.041 ± 0.015 seconds; p = 0.723), and complications. The post-injection mHHS was improved in all three approaches without significant differences.

## Introduction

The use of intra-articular steroid injection of the hip helps differentiate between intra-articular hip pathologies and extra-articular hip pathologies as the causes of hip pain and for the therapeutic management of hip osteoarthritis or synovitis^1–7^. Intra-articular steroid injection has a sensitivity of 91.5% and specificity of up to 100% to identify intra-articular pathology if the patient experienced improvement of symptoms after the procedures^2^.

Currently, the intra-articular hip injection has been widely used, with the three common approaches being the anterior approach (A)^8^, the anterolateral approach (AL)^9^, and the proximal anterolateral approach (PAL)^10^. Despite the large quantities of research surrounding the use of intra-articular steroid injection of the hip, the majority of researchers emphasized the improvement of symptoms, and in comparing the assistive method such as comparing ultrasound-assisted intra-articular hip injection with fluoroscopic assisted intra-articular hip injection. None of the studies focus on comparing the accuracy and efficacy of three common fluoroscopic guidance intra-articular hip injection approaches.

This study aimed to compare the efficacy of anterior, anterolateral, and proximal anterolateral approaches of the fluoroscopy-guided intra-articular hip injection. We hypothesized that there would be a significant difference in efficacy between the three intra-articular steroid injection approaches and that this study would be able to guide the physician in choosing the best approach for their patients.

## Methods

The patient recruitment was performed at Thammasat University Hospital in January 2020 after approval by the ethics committee. All procedures performed in studies involving human participants were approved by the ethics committee of Thammasat University (Registration no. MTU-EC-OT-1-184/62) on 20/01/2020. The study protocol was registered and submitted to the Thai Clinical Trials Registry (TCTR) with the TCTR identification number TCTR20200722002. The date of the first registration was 22/07/2020. All methods were performed in accordance with the relevant guidelines and regulations.

A prospective randomized controlled trial of hip intra-articular injection was started from 01/08/2020 to 31/03/2022 after the protocol had been registered and approved in the clinical trial registry. The inclusion criteria included patients with intra-articular hip disorders from osteoarthritis, femoroacetabular impingement (FAI), synovitis, and labral lesions who indicated an intra-articular steroid injection. The exclusion criteria included significant degenerative spine pathology with radiculopathy or significant neurological deficits, previous hip surgery of the injection side, presence of an osteolytic or osteoblastic lesion on imaging suspected tumor or infection origins, active infection around the hip, allergic to steroid or contrast media, and body mass index (BMI) more than 35 kg/m^2^.

The term “efficacy” in this study was primarily defined as the success of “one attempt of fluoroscopy-guided injection”. The secondary outcomes of efficacy included pain during injection, radiation doses and time, complications, and short-term functional score improvement using the modified Harris hip score (mHHS)^11^.

From the pilot study, the resulting efficacy defined by “single attempt of injection” for the anterior, anterolateral, and proximal anterolateral approaches in 30 patients were 70%, 70%, and 90% respectively. Based on a non-inferiority trail with a 10% non-inferiority limit, the significance level (alpha) was 0.05, and the power was 90%. The output of the sample size calculation from n4Studies was 29 samples per group, with a total of 87 patients.

**Figure 1:**
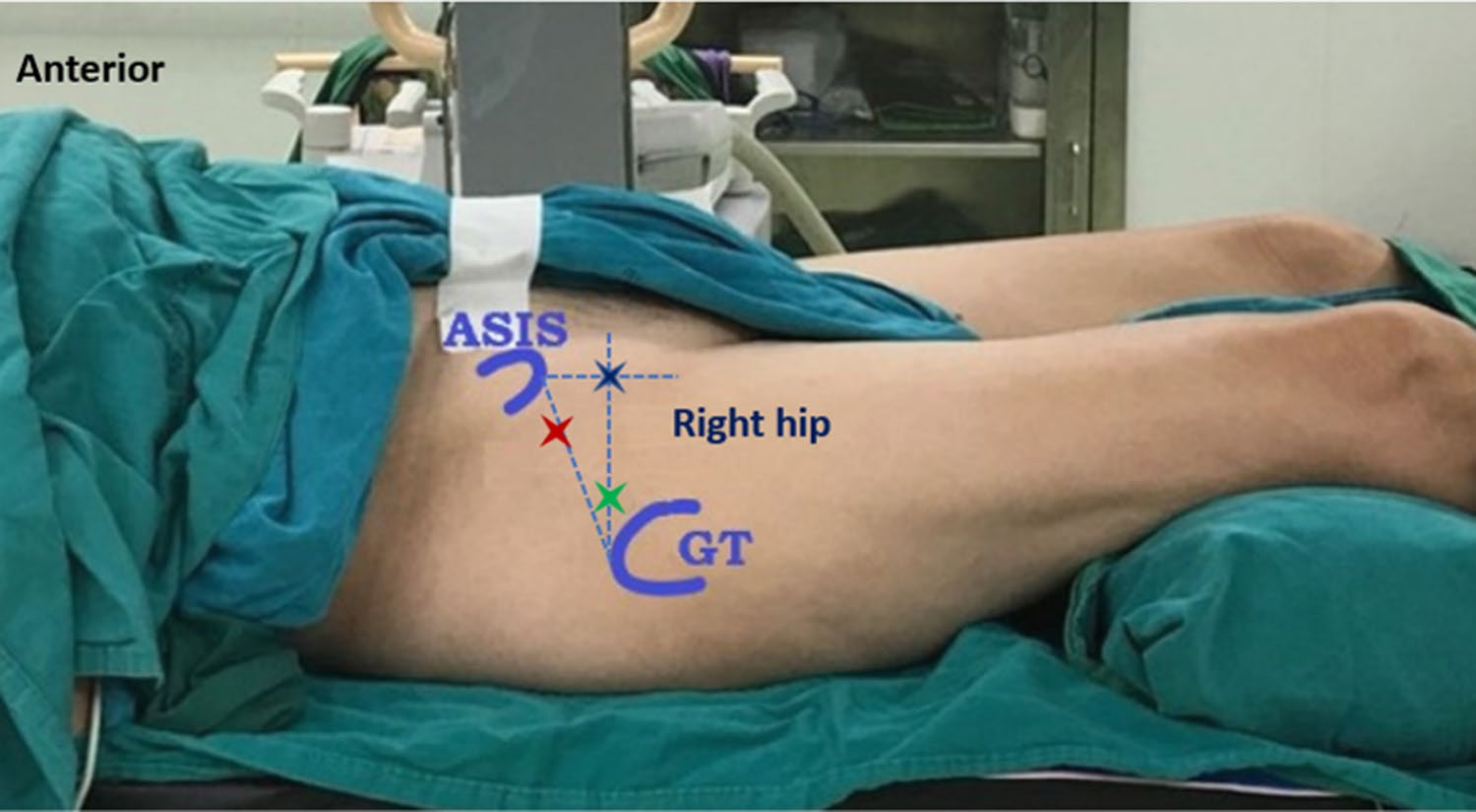
Demonstrated the surface landmark of each injection approach on the right hip. The first line was drawn from ASIS down to the center of the patella (line 1), the second line was drawn from the superior edge of the greater trochanter (GT) to intersect with line 1 perpendicularly (line 2), and the third line was drawn from the anterosuperior iliac spine (ASIS) directed inferolateral to the tip of GT (line 3). The entry point for the anterior approach (A) is the intersection between lines 1 and 2 (blue X mark). The entry point for the anterolateral approach (AL) is about 1 centimeter superior and anterior to the tip of GT (green X mark). The entry point for the proximal anterolateral approach (PAL) is about 1/3 upper portion of line 3 (red X mark).

After patient screening and recruitment by eligible criteria, all patients were advised of the procedures, risks, and benefits of the research, thereafter the patients were informed and signed the consent form. The pre-injection mHHS was evaluated on recruitment day. On the injection day, patients stayed in a supine position and put the pillow underneath the patient’s knees to provide about 20 degrees of hip flexion. The injection site was prepared in a standard sterile fashion. The approach of injection was random using computerized randomization with the block of 6 and allocated with a sealed opaqued envelope before injection. The injection was performed by a single fellowship-trained surgeon in hip arthroscopy under fluoroscopic guidance to enhance visual clarity, in the operation room without the pre-medication. The surface landmark of each injection approach on the right hip is shown in Fig. 1. The first line was drawn from ASIS down to the center of the patella (line 1), the second line was drawn from the superior edge of the greater trochanter (GT) to intersect with line 1 perpendicularly (line 2), and the third line was drawn from the anterosuperior iliac spine (ASIS) directed inferolateral to the tip of GT (line 3). The entry point for the anterior approach (A) is the intersection between lines 1 and 2 (blue X mark). The entry point for the anterolateral approach (AL) is about 1 centimeter superior and anterior to the tip of GT (green X mark). The entry point for the proximal anterolateral approach (PAL) is about 1/3 upper portion of line 3 (red X mark).

The intra-articular injection was performed using needle No.25, and a local anesthetic solution of 1% xylocaine without adrenaline 3 mL was injected. The Visual analog scale (VAS) during local anesthetic injection was evaluated. Then, using spinal needle No. 20 (Length of 90 mm) to inject the hip joint following protocol for each approach, the mixture of “Triamcinolone acetonide (40 mg) 1 mL plus an OmniplaqueTM (350mg/mL) 1 mL plus the 1% Xylocaine without adrenaline 8 mL” were injected into the hip joint. Fluoroscopic imaging (PHILIPS BV Pulsera) was used in the anteroposterior (AP) view to identify whether the approach was successful. “Success” is defined by the contrast media filled inside the hip capsule.

“Single attempt” was defined as the single shot in injection without redirection of the spinal needle (redirection was defined by changing the direction by pulling back the needle more than half of the needle length). “More than one attempt or multiple attempts” was defined as the need for redirection by pulling a spinal needle out for more than 40 mm (half of the needle length). VAS during intra-articular hip injection was recorded.

Radiation exposure was recorded as doses and radiation time. Complication such as infection or vasovagal symptoms was recorded (if occurred). The patients were made appointments for subsequence follow-ups at 2 weeks intervals and were evaluated for post-injection mHHS.

### Statistical analysis

Descriptive data were reported as frequencies (percentage, %) or mean ± standard deviation (SD). The Fisher exact test was used to compare categorical data between groups e.g. gender, and affected side. The Oneway-ANOVA test was used for comparisons of continuous variables with normal distribution, and the Kruskal-Wallis’s test was used for continuous variables with non-normal distribution including age, BMI, VAS during local anesthetic injection, VAS during intra-articular injection, radiation exposure, pre-injection mHHS, post-injection mHHS. P < 0.05 was statistically significant. Data were analyzed using Stata version 14.2 (StataCorp, Lakeway, Texas, USA).

## Results

**Figure 2:**
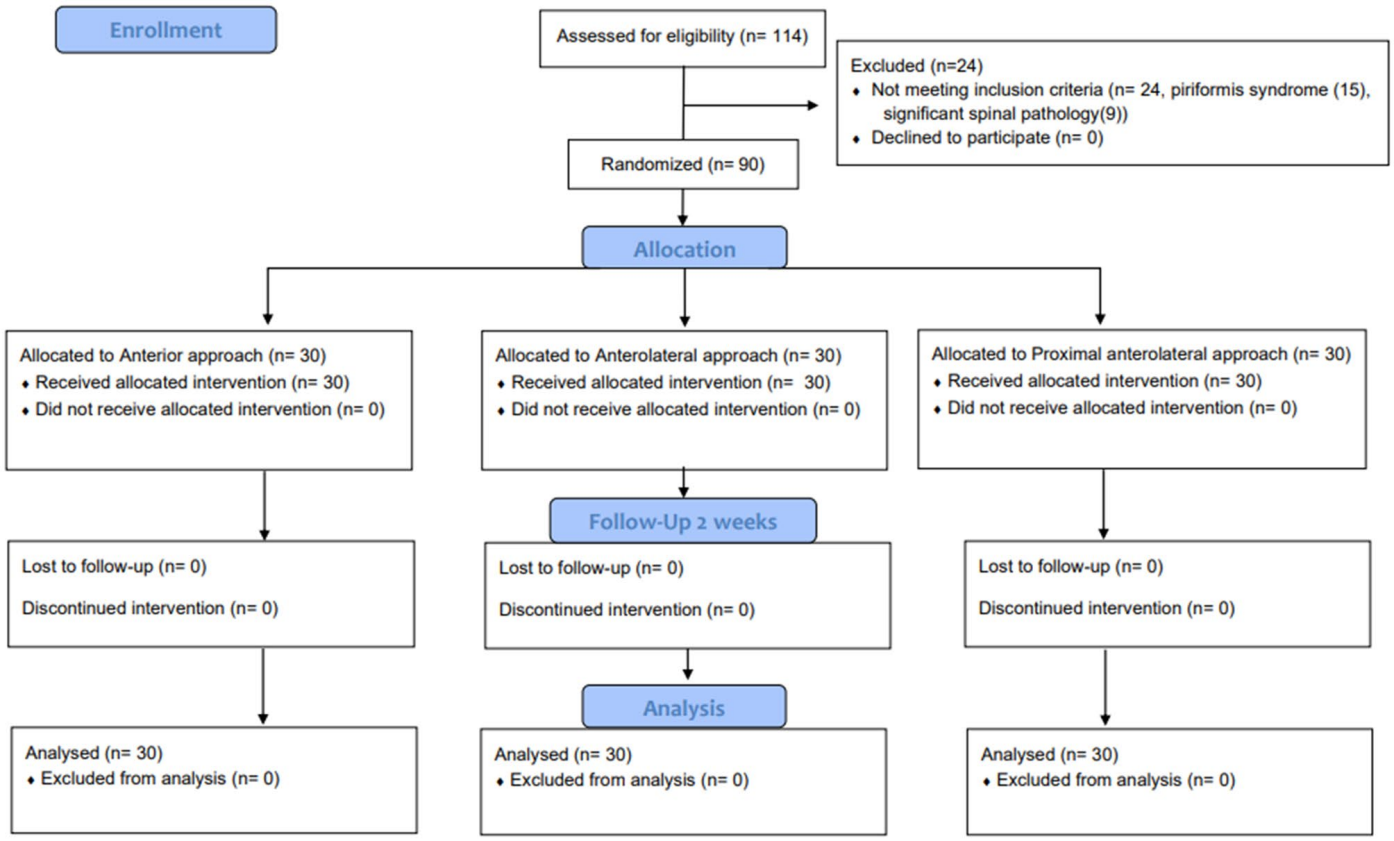
CONSORT flow diagram of the study.

A total of 90 patients were included with 30 patients in each group. The CONSORT flow diagram is presented in Fig. 2.

Patient characteristics are shown in Table 1. The majority of the study population was female (71%) with a mean age of 54.8 years old. Osteoarthritis of the hip was the most common pathology in about 82%. There was no significant difference between the patient demographics who received each injection approach.

**Table 1:** Demographic data of the study population.

Demographic data	Anterior approach (N=30)	Anterolateral approach (N=30)	Proximal anterolateral approach (N=30)	Total (N=90)	*p*-value
Gender					
Female (%)	18 (60%)	22 (73%)	24 (80%)	64 (71%)	0.266*
Male (%)	12 (40%)	8 (27%)	6 (20%)	26 (29%)	
Age (year) (mean ± SD)	58.67 ± 12.48	53.47 ± 18.37	52.27 ± 16.33	54.8 ± 15.98	0.259**
Body weight (kg) (mean ± SD)	66.02 ± 12.18	63.37 ± 13.74	62.85 ± 11.29	64.08 ± 12.38	0.574**
Height (cm) (mean ± SD)	162.57 ± 9.24	160.7 ± 8.39	162.23 ± 8.54	161.83 ± 8.67	0.678**
BMI (kg/m^2^) (mean ± SD)	24.99 ± 4.25	24.41 ± 4.28	23.85 ± 3.68	24.42 ± 4.06	0.562**
Affected side					
Left (%)	14 (47%)	14 (47%)	11 (37%)	39 (43%)	0.697*
Right (%)	16 (53%)	16 (53%)	19 (63%)	51 (57%)	
Diagnosis					
Osteoarthritis (%)	27 (90%)	25 (83%)	22 (73%)	74 (82%)	
FAI (%)	–	2 (7%)	4 (13%)	6 (7%)	
Osteonecrosis (%)	–	1 (3%)	2 (7%)	3 (3%)	
Labral tear (%)	2 (7%)	2 (7%)	1 (3%)	5 (6%)	
Synovitis (%)	1 (3%)	–	1 (3%)	2 (2%)	
Pre-injection mHHS (mean ± SD)	55.51 ± 19.01	59.40 ± 15.60	56.21 ± 17.47	57.04 ± 17.30	0.655**

*BMI* body mass index, *SD* standard deviation, *FAI* femoroacetabular impingement, *mHHS* modified Harris hip score.

*Calculated using Fisher’s exact test.

**Calculated using Oneway-ANOVA.

The success in “single attempt” was 80%, 80%, and 90% in A, AL, and PAL approaches respectively. There was no statistically significant difference in single-attempt success between the three injection techniques (p = 0.533). The accuracy rate of intra-articular injection in the study was 100% in all approaches either single or multiple attempts by the fluoroscopic guidance and controlled injection, confirmed by observing the contrast media filled within the hip joint capsule in all cases. There were no statistically significant differences in the VAS during local anesthetic injection (p = 0.5), VAS during intra-articular injection (p = 0.587), radiation doses (p = 0.788), and radiation time (p = 0.723). The results are shown in Table 2.

**Table 2:** Result of the difference between three approaches to hip injection.

Results	Anterior approach (N=30)	Anterolateral approach (N=30)	Proximal anterolateral approach (N=30)	Total (N=90)	*p*-value
Attempt					
Single attempt (%)	24 (80%)	24 (80%)	27 (90%)	75 (83%)	0.533*
> 1 attempt (%)	6 (20%)	6 (20%)	3 (10%)	15 (17%)	
VAS during local anesthetic injection (mean ± SD)	4.33 ± 1.99	3.70 ± 2.34	4.27 ± 2.49	4.10 ± 2.27	0.500**
VAS during intra-articular injection (mean ± SD)	4.27 ± 1.87	4.70 ± 2.37	4.13 ± 2.37	4.37 ± 2.21	0.587**
Radiation doses (mGy) (mean ± SD)	0.558 ± 0.313	0.526 ± 0.485	0.492 ± 0.275	0.525 ± 0.366	0.788**
Radiation time (seconds) (mean ± SD)	0.043 ± 0.017	0.039 ± 0.021	0.041 ± 0.015	0.041 ± 0.017	0.723**
Complication (%)	–	–	1 (3%)	1 (1%)	
Pre-injection mHHS (mean ± SD)	55.51 ± 19.01	59.40 ± 15.60	56.21 ± 17.47	57.04 ± 17.30	0.655**
Post-injection mHHS (mean ± SD)	86.01 ± 13.35	85.61 ± 12.41	84.91 ± 15.60	85.51 ± 13.70	0.953**

*VAS* visual analog scale, *SD* standard deviation, *mHHS* modified Harris hip score.

*Calculated using Fisher’s exact test.

**Calculated using Oneway-ANOVA.

The overall complication was 1 patient of 90 (1%) in the PAL injection approach, the patient developed vasovagal symptoms after a single attempt of injection which spontaneously resolved after a rest of about 2–3 minutes without the need for further management. There was no patient with injury of the lateral femoral cutaneous nerve of the thigh or infection after injection. For the post-injection mHHS collected 2 weeks after injection, there was an improvement from pre-injection in all approaches.

## Discussion

From this study, using the three approaches, namely anterior, anterolateral, and proximal anterolateral approaches for fluoroscopy-guided intra-articular hip injection, the overall successful intra-articular hip injection in a single attempt is 83%, with the anterior approach in 80%, anterolateral approach in 80%, and proximal anterolateral approach in 90%, we did not find the statistically significant differences between three approaches. The VAS during local anesthetic injection, VAS during intra-articular injection, radiation doses, radiation time of exposure, and the post-injection mHHS were similar in all three approaches without significant differences between groups.

The accuracy rate in our study is 100% (including 83% single attempt, and 17% multiple attempts) intra-articular injection in all approaches during the use of fluoroscopic guidance and controlled injection. After the tip of the needle reached the femoral head-neck area, we found the contrast within the hip joint capsule in all patients, we could not observe any extra-articular leakage of the radio-opaque media. Compared to the previous study on the accuracy of each approach which reported a 93^8^–96.7%^9^ accuracy for the anterior approach, 94–95% accuracy for the proximal anterolateral approach^10,12^, and 100% accuracy for the anterolateral approach^9^. Our study had a high accuracy rate, we hypothesized that this might result from the difference in the experience of performers and the injection techniques in each study. Furthermore, in our injection technique, the hip was about 20 degrees flexion, we would check the direction of the needle using the fluoroscopic control until reached the desired direction to the anterior or anterolateral site of the femoral head-neck junction and the tip of the needle always need to touch the bone before we injected. We injected the steroid and the mixtures by slightly pushing and pulling the needle backward-forward around the point of injection area and controlling with the sequential fluoroscopy. With this technique, we’ve achieved a standardized procedure with reliable results for hip injection.

For the secondary outcome, we report no statistically significant difference in pain using a visual analog scale (VAS), both during local anesthetic injection and intra-articular hip injection between the three approaches, with the mean VAS of about 4 on both procedures. Even though the data focusing on pain during the procedure have not yet been previously reported, from the result of our study which showed a similar level of pain in all approaches, we can conclude that pain should not be a factor when considering the intra-articular hip injection approaches. Additionally, we’ve found no statistically significant difference in radiation exposure between the approaches. The average radiation doses (anterior of 0.56 ± 0.31, anterolateral of 0.53 ± 0.49, and proximal anterolateral of 0.49 ± 0.28 mGy) were less than another study^9^ showed anterior and lateral median radiation doses of 1 mGy and 3 mGy, respectively. The radiation doses in our study are within the standard exposure for radiographs of the pelvis (1.31 mGy)^13^.

Throughout our study, we’ve only experienced one complication from ninety patients, which accounts for 1.1%. Details on the event were as follows, the patient was 29 years 29-year-old man diagnosed with secondary osteoarthritis of the right hip after trauma, he was allocated to the PAL injection group, and during the procedure, he experienced lightheadedness with normal vital signs and oxygen saturation. The symptoms recovered spontaneously after resting for 2 minutes, thus the symptom was considered to be caused by vasovagal symptoms. Following 2 weeks, the patient returned for follow-up with an improvement in mHHS from 58.3 pre-injection to 82.5 post-injection, no further complication was recorded. There were no serious complications^14,15^ related to the injection.

Comparing the pre-injection mHHS with post-injection mHHS, there was no statistically significant difference between the three groups. However, comparing the pre-injection score with post-injection mHHS in the same group, we reported a significant improvement in post-injection mHHS in all three groups. The results represent the improvement in very short-term outcomes of successful intra-articular steroid injection of the hip despite the approach being used. We did not evaluate the outcomes of longer follow-up because of the natural history of the intra-articular steroid injection that mainly affected only about 6–12 weeks after injection^1,16–18^ and we aimed to evaluate the short-term efficacy between the three different hip injection approaches.

Hip injection using anatomic landmarks without ultrasonographic or radiographic assistance is useful and provides acceptable accuracy^12,19,20^. This study used the fluoroscopy-guided hip injection, which is common and standard for the diagnostic and therapeutic management of hip disorder patients^1,7,21,22^.

Our study is the first study to compare the efficacy between three commonly used techniques for intra-articular hip injection. No study compared the safety and outcomes of each injection approach. Additionally, all operating procedures were performed by a single fellowship-trained surgeon in hip arthroscopy, and the operative setting was standardized in every patient therefore the errors and variations in the injection techniques were minimized in our study.

Our study is not without limitations, however. Due to the single well-trained performer in our study, our result may not be generalizable to a general orthopedic surgeon or general practitioners, however in providing detailed instruction on the operative setting and procedure we used in this study, we believed that similar result could be achieved even with less experienced practitioners. Furthermore, our study has a small sample size, which might not represent the general population, and has low power to detect the statistical significance. The very short-term outcomes were evaluated that could not evaluate the effect of each approach in terms of medium- to long-term therapeutic outcomes. Finally, the non-blind nature of our study design could cause bias in the outcome measure.

## Conclusion

Fluoroscopy-guided hip injection via the anterior (A), anterolateral (AL), or proximal anterolateral (PAL) approaches were comparable in efficacy regarding the number of attempts, VAS during injection, radiation exposure, post-injection mHHS, and complications. The physicians can choose any of their preferred fluoroscopy-guided hip injection approaches.

## Data Availability

The datasets used and/or analyzed during the current study are available from the corresponding author upon reasonable request.
